# Glomerular disease search filters for Pubmed, Ovid Medline, and Embase: a development and validation study

**DOI:** 10.1186/1472-6947-12-49

**Published:** 2012-06-06

**Authors:** Ainslie M Hildebrand, Arthur V Iansavichus, Christopher WC Lee, R Brian Haynes, Nancy L Wilczynski, K Ann McKibbon, Michelle A Hladunewich, William F Clark, Daniel C Cattran, Amit X Garg

**Affiliations:** 1 Division of Nephrology, University of Western Ontario, London, Canada; 2 Department of Clinical Epidemiology and Biostatistics, McMaster University, Hamilton, Canada; 3 Department of Medicine, McMaster University, Hamilton, Canada; 4 Division of Nephrology, University of Toronto, Toronto, Canada; 5 Department of Epidemiology and Biostatistics, University of Western Ontario, London, Canada; 6 London Kidney Clinical Research Unit, Room ELL-101, Westminster, London Health Sciences Centre, 800 Commissioners Road East, London, Ontario, N6A 4 G5, Canada

**Keywords:** Glomerular diseases, Glomerulopathy, Medical Informatics, Information retrieval, Medline, Embase

## Abstract

**Background:**

Tools to enhance physician searches of Medline and other bibliographic databases have potential to improve the application of new knowledge in patient care. This is particularly true for articles about glomerular disease, which are published across multiple disciplines and are often difficult to track down. Our objective was to develop and test search filters for PubMed, Ovid Medline, and Embase that allow physicians to search within a subset of the database to retrieve articles relevant to glomerular disease.

**Methods:**

We used a diagnostic test assessment framework with development and validation phases. We read a total of 22,992 full text articles for relevance and assigned them to the development or validation set to define the reference standard. We then used combinations of search terms to develop 997,298 unique glomerular disease filters. Outcome measures for each filter included sensitivity, specificity, precision, and accuracy. We selected optimal sensitive and specific search filters for each database and applied them to the validation set to test performance.

**Results:**

High performance filters achieved at least 93.8% sensitivity and specificity in the development set. Filters optimized for sensitivity reached at least 96.7% sensitivity and filters optimized for specificity reached at least 98.4% specificity. Performance of these filters was consistent in the validation set and similar among all three databases.

**Conclusions:**

PubMed, Ovid Medline, and Embase can be filtered for articles relevant to glomerular disease in a reliable manner. These filters can now be used to facilitate physician searching.

## Background

Retrieving health literature is the cornerstone to evidence-based practice. However, the sheer volume of available information presents a challenge to even the most skilled physicians and researchers. Many users lack knowledge of information sources, have difficulty formulating an optimal search strategy, and are short on time [1-3]. These obstacles may be even greater when dealing with an area of nephrology such as glomerular disease, which is particularly broad, multidisciplinary, and difficult to define. Indexing of articles is often inconsistent, with variable terminology used for similar clinical entities or histologic diagnoses [4]. In this case, searches need to be highly sensitive to ensure important evidence is not overlooked, while minimizing the retrieval of non-relevant articles to ensure efficiency.

Search filters are a logical way to deal with these barriers. Filters are pre-tested searches created by strategically combining individual and combinations of search terms to achieve optimal article retrieval for a given purpose. Many filters already exist including those optimized to retrieve studies and systematic reviews of diagnosis, etiology, treatment, outcomes, adverse events, prognosis, and clinical prediction guides [5-14]. More recently, topic-based search filters have started to emerge [15-17]. Within the area of nephrology, search filters already exist to retrieve renal information and articles relevant to kidney transplantation [15,18]. However, none of these filters were designed to enhance retrieval of articles relevant only to glomerular disease.

A search filter for glomerular disease would allow physicians to perform searches within a subset of articles in an online database that were preselected as relevant to this content area. For example, if a user wanted to determine the most effective immunosuppressive therapy for a case of membranous nephropathy, they could combine the terms ‘treatment membranous’ with the glomerular disease search filter to improve the precision of article retrieval. The search filter acts as an optimized substitute for topic-specific terms required to increase the sensitivity and specificity of the search and eliminates the need to enter these glomerular disease terms and synonyms in the search query (e.g., nephropathy, glomerulopathy, glomerulonephritis). This strategy, in theory, should maximize the retrieval of articles relevant to glomerular disease and minimize non-relevant articles, increasing the overall precision of each search.

We conducted this study to develop and test glomerular disease search filters for PubMed, Ovid Medline, and Embase. Afterwards, we did some proof of concept searches to illustrate the potential effectiveness of these new filters with real physician searches in the PubMed database at large.

## Methods

We used a diagnostic test assessment framework to develop and validate search filters for glomerular disease. For the purpose of this study, glomerular disease was defined as any disease in which the glomerulus of the kidney is affected, resulting in hyperplasia, atrophy, necrosis, scarring, or deposits in the glomeruli.

### Sample of articles

We first established the reference standard by manual review of all full text articles published in 39 journals from 2004 to 2008. To develop this collection of journals, we adopted a similar strategy for article sampling as published in prior search filter studies. This approach has resulted in filters that generalize well over publication years and journal types [15,19,20]. We compiled a list of 466 journals from a list of journals that had published at least one article relevant to renal care from 1961 to 2005 [21]. We then ranked these journals according to the number of articles with relevant information and selected the top 20 journals. In addition to this, we selected 19 more journals at random from the remaining 446 journals. We then randomly divided these 39 journals into development and validation sets at a ratio of two to one respectively.

### Article review

We manually reviewed all full text articles indexed in PubMed, Ovid Medline, and Embase from 2004 to 2008 for each journal in the development and validation set for relevance to glomerular disease ( Additional file 1: Appendix A). These 22,992 articles included original investigations, reviews, letters, and editorials. We derived a standardized checklist of qualifications and terms to classify articles as relevant to glomerular disease from a review of nephrology textbooks and the MeSH thesaurus ( Additional file 1: Appendix B). Three readers (AI, CL, AG) used this checklist to determine whether the full text of each article was relevant to nephrology. All reviewers were calibrated against a nephrologist (AG) in their application of checklist criteria using two test sets of 100 articles (agreement beyond chance, κ = 0.91).

### Filters

We developed unique filters for PubMed, Ovid Medline, and Embase. We obtained the search terms used for filter development from the following sources: US National Library of Medicine (NLM) medical subject heading (MeSH) thesaurus using Medline MeSH browser [22], Medline permuted index [23], Emtree thesaurus [24], SNOMED clinical terms, nephrology textbooks [25], clinical practice guidelines [26,27], systematic reviews [28-33], website glossaries, and clinician and librarian opinion. All terms considered potentially useful by any member of our team were included. Examples of terms used in the filters include ‘glomerulonephritis’, ‘proteinuria’, ‘nephrotic’, and ‘biopsy’. We used MeSH terms with or without major focus and with or without additional subheadings or explosion capability. Major focus refers to records in which an index term has been tagged as the major topic of the article. Entering the exploded MeSH term ‘glomerulonephritis’ means the following terms are also automatically included in the search: anti-glomerular basement membrane disease, IgA, membranoproliferative, membranous, focal segmental glomerulosclerosis, and lupus nephritis. We considered free text words as full and truncated terms and accounted for both American and British English spelling. The inclusion of multiple endings was achieved through the use of the $ symbol (for example, glomerulo$). Terms could appear anywhere in a citation, but not solely in the journal name. We repeated the same process for Embase using EMTREE index terms to replace the MeSH terms in PubMed and Ovid Medline.

We automated the process of combining and testing the filters by using a computer-implemented algorithm. We combined single term filters into multiple term filters by selectively using the Boolean operators “OR,” “AND,” and “NOT” to maximize sensitivity and specificity. We then compared the retrieval performance of various filters (made up of individual and combinations of search terms) with the reference standard from manual review in the development set.

### Statistical analysis

For each filter, we constructed a two by two contingency table and assessed filter performance by calculating sensitivity, specificity, precision, and accuracy, similar to evaluation of a diagnostic test (Table 1). We then selected filters from the development phase that demonstrated high performance in either sensitivity or specificity without compromising precision and retested them in the validation set of articles.

**Table 1:** Two by two contingency table comparing filter to ‘reference standard’

**Filter (consisting of single or combined terms)**	**Manual review of each article**	
	**Articles relevant to glomerular disease**	**Articles not relevant to glomerular disease**
Article identified	a	b
Article not identified	c	d

Sensitivity = a/(a + c): proportion of all articles with information on glomerular disease in the reference set that are retrieved by the filter (also called recall in information retrieval studies).

Specificity = d/(b + d): proportion of all articles without information on glomerular disease in the reference set that are correctly not retrieved by the filter.

Precision = a/(a + b): proportion of all articles retrieved by the filter with information on glomerular disease (also referred to as positive predictive value in diagnostic test terminology).

Accuracy = (a + d)/(a + b + c + d): proportion of all articles dealt with correctly by filter.

### Proof of concept searches

To illustrate the potential effectiveness of validated filters in PubMed, we selected six independent nephrologists from a directory of Canadian nephrologists provided by the Royal College of Physicians and Surgeons of Canada to execute a search for a unique predetermined clinical question. We formulated six clinical questions, each which could be answered by a recent corresponding systematic review [28-33]. These systematic reviews were then used as a reference source for relevant articles on the given topic. For example, the question ‘What are the benefits and harms of different interventions for the treatment of renal vasculitis in adults?’ was framed to match a systematic review of thirteen articles on interventions for renal vasculitis in adults by Walters et al. [32]. We asked each nephrologist to formulate a search strategy for the given clinical question without knowledge of the search filter or database in use. We then applied these searches to the PubMed database with and without the validated filters developed as part of this study. Search dates were restricted to the date on which the review was updated. In each case, we noted the number of relevant articles identified in searches with and without the validated filters, compared with the reference standard, which in this case was the set of relevant articles as determined by each systematic review.

## Results

### Sample of articles

We used 22,992 full text articles from 39 journals ( Additional file 1: Appendix A). In total, 21,300 articles contributed to the PubMed set, while 21,280 and 22,158 articles contributed to the Ovid Medline and Embase set, respectively. We assigned 14,619 articles to the development set and 8,373 articles to the validation set.

### Single term filters

We tested 261,255 single term filters. The single term filters with optimal balance of sensitivity and specificity in the development set were ‘Kidney Diseases[mh]’ for PubMed (90.2% sensitivity, 87.0% specificity), ‘exp Kidney Diseases/’ for Ovid Medline (90.2% sensitivity, 87.0% specificity), and ‘exp kidney disease/’ for Embase (95.3% sensitivity, 80.6% specificity).

### Multiple term filters

We tested 736,043 multiple term filters. Our best performing filters for PubMed, Ovid Medline, and Embase are shown in Table 2, categorized by high-sensitivity and high-specificity. These filters used over 50 terms. All filters in the development set achieved 93.8-99.0% sensitivity, 95.2-98.6% specificity, 43.4-71.1% precision, and 95.3-98.5% accuracy. Filters optimized for sensitivity achieved 96.7-99.0% sensitivity in the development set and filters optimized for specificity achieved 98.4-98.6% specificity (Table 2).

**Table 2:** Glomerular Disease Search Filters for PubMed, Ovid Medline, and Embase Optimized for High Sensitivity and High Specificity

		**Set**	**Sensitivity (%)**	**Specificity (%)**	**Precision (%)**	**Accuracy (%)**
			**(95% CI)**	**(95% CI)**	**(95% CI)**	**(95% CI)**
PubMed Filters^‡^						
High-Sensitivity Filter	(nephropath*[tw] OR glomerulonephrit*[tw] OR proteinuri*[tw] OR nephrotic[tw] OR glomerulosclerosis[tw] OR nephrit*[tw] OR kidney biopsy[tiab] OR renal biopsy[tiab] OR albuminuri*[tw] OR glomerulopath*[tiab] OR membranoproliferative[tw] OR mesangioproliferative[tiab] OR nephrosis[tw] OR microalbuminuri*[tiab] OR diabetic kidney[tw] OR diabetic renal[tw] OR anti-glomerular[tw] OR nephrosclerosis[tiab] OR alport*[tw] OR goodpasture*[tiab] OR minimal change[tiab] OR glomerular disease*[tiab] OR anca*[tw] OR anti-neutrophil[tw] OR polyangiitis[tw] OR polyangitis[tw] OR antineutrophil[tw] OR antiglomerular[tw] OR "Kidney Glomerulus"[majr:noexp] OR "Lupus Erythematosus, Systemic"[majr:noexp] OR "Vasculitis"[majr:noexp] OR "Purpura, Schoenlein-Henoch"[mh:noexp] OR "Anti-Neutrophil Cytoplasmic Antibody-Associated Vasculitis"[mh] OR ((lupus[tw] OR vasculitis[tw] OR purpura[tw] OR granulomatosis[tw]) AND (glomerul*[tw] OR kidney[tw] OR renal[tw] OR nephrolog*[tw])) OR ((chronic kidney[tiab] OR chronic renal[tiab] OR "Renal Insufficiency, Chronic"[mh]) AND ("Diabetes Mellitus, Type 2"[majr:noexp] OR "Kidney Glomerulus"[mh:noexp])) OR ("Kidney Diseases"[majr] AND (biopsy[tw] OR myeloma[tiab] OR renin-angiotensin[tw] OR hiv[tiab] OR amyloid*[tw])))	Development	96.7 (95.1-98.3)	95.2 (94.9-95.6)	43.4 (40.4-46.3)	95.3 (94.9-95.7)
		Validation	94.8 (91.1-98.6)	96.0 (95.5-96.4)	28.5 (24.3-32.7)	95.9 (95.5-96.4)
High-Specificity Filter	(glomerulonephrit*[tw] OR nephrotic[tw] OR diabetic nephropath*[tw] OR glomerulosclerosis[tw] OR iga nephropath*[tiab] OR minimal change[tiab] OR membranoproliferative[tw] OR glomerulopath*[tiab] OR membranous nephropathy[tiab] OR antineutrophil[tw] OR nephrosis[tw] OR anca*[tw] OR diabetic kidney[tiab] OR anti-glomerular[tw] OR glomerular disease*[tiab] OR anti-neutrophil[tw] OR antiglomerular[tw] OR polyangiitis[tw] OR alport*[tw] OR mesangioproliferative[tiab] OR goodpasture*[tiab] OR immunoglobulin a nephropathy[tiab] OR "AIDS-Associated Nephropathy"[majr:noexp] OR "Purpura, Schoenlein-Henoch"[majr:noexp] OR "Nephritis, Hereditary"[majr:noexp] OR "Anti-Neutrophil Cytoplasmic Antibody-Associated Vasculitis"[majr] OR "Balkan Nephropathy"[majr:noexp] OR (diabet*[ti] AND nephropath*[ti]) OR ((kidney[tw] OR renal[tw] OR nephrit*[tw] OR nephrolog*[tw] OR glomerul*[tw]) AND (purpura[tw] OR lupus[tw] OR vasculitis[tw])) OR ((nephrit*[tw] OR "Renal Insufficiency, Chronic"[mh] OR chronic kidney[tiab] OR chronic renal[tiab]) AND ("Kidney Glomerulus"[mh:noexp] OR "Diabetes Mellitus, Type 2"[majr:noexp] OR microalbuminuri*[tiab] OR immunoglobulin[tiab])) OR ((proteinuri*[ti] OR "Proteinuria"[majr:noexp]) AND (hematuria[tw] OR haematuria[tiab] OR glomerulo*[tw] OR amyloid*[tw] OR albuminuri*[tiab] OR inflammation[tiab] OR myeloma[tw])) OR ((glomerulo*[tw] OR nephropath*[tiab] OR proteinuri*[ti] OR microalbuminuri*[ti] OR albuminuri*[ti]) AND ("Diabetes Mellitus, Type 2"[majr:noexp] OR "Diabetic Angiopathies"[majr] OR overt[tiab])) OR ("Kidney Diseases"[majr:noexp] AND (amyloid*[tw] OR mesangio*[tiab] OR sclerosis[tiab])))	Development	93.8 (91.6-95.9)	98.4 (98.2-98.6)	69.2 (65.6-72.7)	98.3 (98.0-98.5)
		Validation	91.1 (86.3-95.9)	98.5 (98.2-98.7)	50.4 (44.1-56.7)	98.4 (98.1-98.6)
Medline Filters^†^						
High-Sensitivity Filter	(nephropath$.mp OR glomerulonephrit$.mp OR proteinuri$.mp OR nephrotic.mp OR glomerulosclerosis.mp OR nephrit$.mp OR renal biopsy.tw OR albuminuri$.mp OR minimal change.tw OR glomerulopath$.tw OR membranoproliferative.mp OR antineutrophil.mp OR glomerular disease$.tw OR nephrosis.mp OR microalbuminuri$.tw OR anca$.mp OR (diabetic adj (kidney or renal)).mp OR kidney biopsy.tw OR anti-glomerular.mp OR antiglomerular.mp OR anti-neutrophil.mp OR polyang?itis.mp OR alport$.mp OR mesangioproliferative.tw OR goodpasture$.tw OR nephrosclerosis.tw OR *Kidney Glomerulus/ OR *Lupus Erythematosus, Systemic/ OR *Vasculitis/ OR Purpura, Schoenlein-Henoch/ OR exp Anti-Neutrophil Cytoplasmic Antibody-Associated Vasculitis/ OR ((glomerul$.mp OR kidney.mp OR renal.mp OR nephrolog$.mp) AND (lupus.mp OR purpura.mp OR vasculitis.mp OR granulomatosis.mp)) OR ((chronic adj2 (kidney or renal)).mp AND (*Diabetes Mellitus, Type 2/ OR Kidney Glomerulus/)) OR (exp *Kidney Diseases/ AND (biopsy.mp OR myeloma.tw OR renin-angiotensin.mp OR hiv.tw OR amyloid$.mp)))	Development	96.7 (95.1-98.3)	95.2 (94.9-95.6)	43.4 (40.4-46.3)	95.3 (94.9-95.6)
		Validation	94.8 (91.1-98.6)	96.0 (95.5-96.4)	28. 6 (24.4-32.8)	96.0 (95.5-96.4)
High-Specificity Filter	(glomerulonephrit$.mp OR (diabetic adj (nephropath$ or kidney)).mp OR nephrotic.mp OR glomerulosclerosis.mp OR iga nephropath$.tw OR minimal change.tw OR glomerulopath$.tw OR membranous nephropathy.tw OR membranoproliferative.mp OR antineutrophil.mp OR glomerular disease$.tw OR nephrosis.mp OR anca$.mp OR anti-glomerular.mp OR anti-neutrophil.mp OR antiglomerular.mp OR polyang?itis.mp OR alport$.mp OR mesangioproliferative.tw OR goodpasture$.tw OR immunoglobulin a nephropathy.tw OR *AIDS-Associated Nephropathy/ OR *Purpura, Schoenlein-Henoch/ OR *Nephritis, Hereditary/ OR exp *Anti-Neutrophil Cytoplasmic Antibody-Associated Vasculitis/ OR *Balkan Nephropathy/ OR (diabet$ AND nephropath$).ti OR ((glomerul$.mp OR kidney.mp OR renal.mp OR nephrit$.mp OR nephrolog$.mp) AND (lupus.mp OR vasculitis.mp OR purpura.mp)) OR ((nephrit$ OR (chronic adj2 (kidney or renal))).mp. AND (Kidney Glomerulus/ OR *Diabetes Mellitus, Type 2/ OR microalbuminuri$.tw. OR immunoglobulin.tw.)) OR ((proteinuri$.ti OR *Proteinuria/) AND (glomerulo$.mp OR h?ematuria.mp OR albuminuri$.tw OR inflammation.tw OR amyloid$.mp OR myeloma.mp)) OR ((glomerulo$.mp OR nephropath$.tw OR proteinuri$.ti OR microalbuminuri$.ti OR albuminuri$.ti) AND (*Diabetes Mellitus, Type 2/ OR exp *Diabetic Angiopathies/ OR overt.tw)) OR (*Kidney Diseases/ AND (amyloid$.mp OR mesangio$.tw OR sclerosis.tw)))	Development	93.8 (91.6-95.9)	98.4 (98.2-98.6)	69.2 (65.6-72.7)	98.3 (98.0-98.5)
		Validation	91.1 (86.3-95.9)	98.5 (98.2-98.8)	50.6 (44.3-56.9)	98.4 (98.1-98.6)
Embase Filters^∫^						
High-Sensitivity Filter	(exp glomerulopathy/ OR ((kidney or renal) adj biopsy).mp OR (diabet$ adj (kidney or renal or nephr$)).mp OR nephrotic.tw OR nephrotic syndrome/ OR glomerulonephrit$.tw OR immunoglobulin a nephropathy/ OR glomerul$ basement membrane$.mp OR iga nephropath$.tw OR minimal change.mp OR membranoproliferative.mp OR (glomerul$ adj (nephr$ or disease$ or scleros$)).tw OR membranous nephr$.tw OR anca$.mp OR proliferative glomerulonephritis/ OR wegener granulomatosis/ OR antineutrophil.mp OR polyang?itis.mp OR *glomerulus/ OR anaphylactoid purpura/ OR alport$.mp OR hiv-associated nephropath$.tw OR anti-neutrophil.mp OR mesangioproliferative.mp OR goodpasture$.mp OR (balkan adj2 nephr$).mp OR heymann nephritis/ OR ((lupus.mp OR vasculit$.mp OR purpur$.mp OR hiv$.tw OR granulomatosis.mp) AND ("Urology and nephrology".ec OR proteinuri$.mp OR nephrit$.mp)) OR ((exp *diabetes mellitus/ OR diabet$.ti) AND (nephropath$.tw OR exp *proteinuria/ OR renin-angiotensin$.mp OR dialys$.tw OR *kidney failure/ OR ((kidney OR renal) adj disease$).ti OR h?emodialys$.tw)) OR (exp *kidney disease/ AND (nephropath$.ti OR exp *proteinuria/ OR renin-angiotensin$.mp OR proteinuri$.ti OR amyloid$.mp)) OR ((exp proteinuria/ OR proteinuri$.mp) AND (nephropath$.tw OR nephrit$.mp OR renin-angiotensin$.tw)))	Development	9.0 (98.1-99.9)	95.3 (94.9-95.6)	43.4 (40.5-46.3)	95.4 (95.1-95.8)
		Validation	96.4 (93.3-99.5)	96.0 (95.6-96.4)	29.4 (25.2-33.4)	96.0 (95.6-96.4)
High-Specificity Filter	(exp *glomerulopathy/ OR nephrotic.tw OR glomerulonephrit$.tw OR diabetic nephropath$.tw OR *diabetic nephropathy/ OR glomerul$ basement membrane$.mp OR nephrotic syndrome/ OR *immunoglobulin a nephropathy/ OR iga nephropath$.tw OR minimal change.mp OR membranoproliferative.tw OR membranous nephropathy.tw OR anca$.mp OR proliferative glomerulonephritis/ OR antineutrophil.mp OR polyang?itis.mp OR *glomerulus/ OR alport$.mp OR hiv-associated nephropath$.tw OR anti-neutrophil.mp OR *anaphylactoid purpura/ OR *wegener granulomatosis/ OR goodpasture$.tw OR mesangioproliferative.tw OR (balkan adj2 nephr$).tw OR ((lupus.mp OR vasculit$.tw OR granulomatosis.mp OR purpur$.tw OR amyloid$.tw) AND (kidney biopsy/ OR proteinuri$.mp OR nephrit$.mp OR serum creatinine.tw)) OR ((exp *diabetes mellitus/ OR proteinuri$.ti OR *proteinuria/) AND (exp glomerulopathy/ OR nephropath$.tw OR *kidney failure/ OR ((kidney or renal) adj disease$).ti OR *microalbuminuria/)) OR ((exp glomerulopathy/ OR diabetic nephropath$.mp) AND (kidney biopsy/ OR mesangial$.tw OR angiotensin$.tw OR serum creatinine.tw OR albuminuri$.mp OR microalbuminuri$.mp)))	Development	95.7 (93.9-97.5)	98.6 (98.4-98.8)	71.1 (67.6-74.6)	98.5 (98.3-98.7)
		Validation	92.8 (88.5-97.1)	98.6 (98.3-98.8)	52.9 (46.6-59.1)	98.5 (98.2-98.7)

‡ PubMed fields: * = truncation character; [tw] = text word present in title, abstract, or MeSH term; [tiab] = term present in title or abstract; [majr:noexp] = not exploded and focused MeSH term; [mh:noexp] = non-exploded MeSH term; [mh] = exploded MeSH term; [majr] = exploded and focused MeSH term; [ti] = term present in title.

† Medline fields: $ = truncation character; mp = multiple posting (term appears in title, abstract, or MeSH); tw = text word present in title; /=MeSH character; adj = adjacent operator; * = focused MeSH term; adj2 = defined adjacency operator; exp = exploded MeSH term; ? = optional wildcard; ti = term present in title.

∫ Embase fields: exp = exploded EMTREE term; /=EMTREE character; adj = adjacent operator; $ = truncation character; mp = multiple posting (term appears in title, abstract, or EMTREE); tw = term present in title or abstract; * = focused EMTREE term; adj2 = defined adjacency operator; ec = Embase section headings field; ti = term present in title.

The performance of these filters was consistent in the validation set. All filters in the validation set achieved 91.1-96.4% sensitivity, 96.0-98.6% specificity, and 95.9-98.5% accuracy, however the precision dropped to 28.5-52.9%. Filters optimized for sensitivity achieved 94.8-96.4% sensitivity in the validation set and filters optimized for specificity achieved 98.5-98.6% specificity (Table 2).

### Proof of concept searches

Selected systematic reviews included a range of 3 to 15 relevant articles. Search phrases determined and entered by the physicians included ‘(mycophenolate OR cyclophosphamide) lupus’, ‘treatment membranous’, ‘steroid HSP’, ‘renal vasculitis treatment limit to English, core journals, adults’, ‘low protein diet diabetes’, and ‘minimal change treatment’. In all proof of concept searches using the validated high-sensitivity filter, the number of non-relevant articles was minimized without compromising the retrieval of relevant articles (Table 3). In proof of concept searches using the validated high-specificity filter, there was a more dramatic reduction in non-relevant articles, however in one case one relevant article was not retrieved using the search ‘low protein diet diabetes’ (Table 3).

**Table 3:** Proof-of-concept searches showing the number of relevant articles retrieved with and without glomerular disease filter

Clinical Question^*^	**Physician search alone**		**Physician search with the high-sensitivity filter**		**Physician search with the high-specificity filter**	
	**Number of non-relevant articles retrieved**	**Number of relevant articles retrieved**	**Number of non-relevant articles retrieved**	**Number of relevant articles retrieved**	**Number of non-relevant articles retrieved**	**Number of elevant articles retrieved**
What are the efficacy and safety of mycophenolate mofetil (MMF) and cyclophosphamide (CYC) in the treatment of proliferative lupus glomerulonephritis (GN)? (7 relevant articles) [29]	2006	7	1721	7	1080	7
Is immunosuppressive treatment effective and safe in the treatment of idiopathic membranous nephropathy (IMN) in adults with nephrotic syndrome? (15 relevant articles) [30]	87500	15	3231	15	2687	15
Does corticosteroid therapy ameliorate the acute manifestations of Henoch-Schonlein purpura or mitigate renal injury? (15 relevant articles) [31]	393	5	68	5	65	5
What are the benefits and harms of different interventions for the treatment of renal vasculitis in adults? (13 relevant articles) [32]	34741	8	8455	8	5923	8
What is the effect of a low-protein diet (LPD) on renal function in patients with type 1 or 2 diabetic renal diseases? (8 relevant articles) [33]	868	7	207	7	180	6
What are the benefits and harms of interventions for the nephrotic syndrome in adults caused by minimal change disease? (3 relevant articles) [28]	4662	3	1236	3	1203	3

* The search phrases as determined and typed in by physicians were: ‘(mycophenolate OR cyclophosphamide) lupus’, ‘treatment membranous’, ‘steroid HSP’, ‘renal vasculitis treatment limit to english, core journals, adults’, ‘low protein diet diabetes’, and ‘minimal change treatment’.

## Discussion

Building on the same concepts our group has used to create novel high performance search filters for general nephrology and renal transplantation [15,18], we have succeeded in developing and validating search filters for glomerular disease that are highly sensitive and specific. All filters achieved a balance of at least 93.8% sensitivity and specificity. Our best performing high-sensitivity filter was in Embase, achieving 99.0% sensitivity and 95.3% specificity. The best performing high-specificity filter was also in Embase, which reached 95.7% sensitivity and 98.6% specificity. Without changing their PubMed search terms, in an illustrative example physicians were able to retrieve articles with a higher degree of precision (less non-relevant articles) with use of these filters.

These filters are complex, often combining in excess of 50 terms with Boolean operators. Coding these filters into the PubMed and Ovid search engine interfaces will permit their easy use by anyone doing a search. In the meantime, we provide these filters at the following link: http://hiru.mcmaster.ca/hiru/hiru_hedges_nephrology_filters.aspx. As of September 2011, use of the high-sensitivity glomerular disease filter reduced the PubMed database from 21 million to 195,374 articles, and the high specificity filter reduced this to 107,658 articles.

Depending on the search terms entered by the user, these filters may serve many purposes, which are best understood in the context of our illustrative proof of concept searches (Table 3). First, without changing the original search term(s), selecting a filter applies the search only to a subset of articles that are richer in glomerular disease content. The result is an increase in precision of the search, similar to the increase in positive predictive value of a screening test when applied to a high-risk population. This was demonstrated by the use of search terms ‘minimal change treatment’ in Table 3. Fewer non-relevant articles were retrieved with use of the filter (1236 versus 4662 articles), without impacting relevant article retrieval. Second, the filter acts as an optimized substitute for glomerular disease specific terms and synonyms allowing users to simplify the search query. This avoids unnecessarily limiting the search due to indexing inconsistencies inherent with the terminology used to define glomerular disease. For example, if a user was searching for dietary recommendations in diabetic nephropathy, the search terms may be simplified to ‘low protein diet diabetes’, instead of searching for ‘low protein diet diabetes’ with selected terms such as ‘nephropathy’, ‘kidney disease’, or ‘glomerulosclerosis’ that may negatively impact relevant article retrieval. In this case, even without use of search terms pertaining to glomerular disease, precision of the results was enhanced (Table 3). Third, users may opt to exclude disease specific terms entirely and use the filter to address questions that potentially relate to all glomerular disease equally. An example of this may include entering ‘immunization’ when addressing the impact of vaccinations in patients with glomerular disease.

Our results also highlight that even with high performance validated search filters, a single search will rarely retrieve everything of relevance on a particular topic. There is simply too much variation in the quality of accompanying search terms entered by the user, completeness of the database, and quality and consistency of indexing. This explains why in some proof of concept searches, retrieval of relevant articles was incomplete both with and without use of the filter (Table 3). Also, the extent to which the search filter is generalizable depends upon the sample of journals selected for study and the method by which articles were defined as relevant. Our selection of journals was deliberately enriched with leading clinical nephrology journals. Although it also included a random sampling of other journals, this set of journals may not adequately represent the complete set of multi-disciplinary journals that feature glomerular disease content in PubMed. This may explain the significant drop in precision when the filter was applied to the validation set, which was a smaller database by design with a lower proportion of relevant articles. Our choice to divide articles into the development and validation sets at the journal level may have also contributed to the lower proportion of articles with glomerular disease content in the validation set. However, this approach provided insight as to what would occur if the search database were expanded to include the over 5000 journals indexed in PubMed.

Proof of concept searches were used to illustrate the functionality of our best performing filters with real physician searches. In each case, the clinical questions formulated from recent systematic reviews were relevant to glomerular disease and physician searches appear typical for the average user. These examples show a gain in search strategy precision with use of the high-sensitivity and high-specificity filter through a dramatic reduction in non-relevant articles. This occurs without sacrificing retrieval of relevant articles in most cases. However, the methods for defining the reference standard based on articles used in systematic reviews of variable quality is indirect and has not been compared with one derived from hand searching [34]. For this reason the proof of concept searches should be viewed as illustrative examples, not as evidence of further filter validation.

These search filters for glomerular disease were designed to offer physicians and researchers a strategy to optimize results by sensitivity or specificity, depending on the level of article retrieval they deem manageable on a practical level. Filters that maximize sensitivity involve a compromise on the level of precision achieved, though this still may appeal to a researcher conducting a systematic review. For busy physicians at the point of care, we recommend starting with the high-specificity filter. To narrow results even further, physicians may prefer use these search filters in conjunction with previously developed methods filters, such as the therapy filter for randomized controlled trials available via PubMed’s Clinical Queries section [5-14]. This approach has not been formally tested with the glomerular disease filters, but in a recent study has been shown to increase the efficiency of retrieval of articles relevant to renal care [35].

## Conclusions

In conclusion, we have succeeded in developing and validating high performance search filters for glomerular disease that can be easily applied by the busy physician. We expect this will contribute to more efficient and effective evidence-based decision-making, education, and patient care. Future research is required to measure this impact, and to better understand the usefulness of these filters when used in combination with previously developed methods filters for physician searches.

## Competing interests

We have no competing interests to declare.

## Authors’ contributions

AMH, AVI, and AXG were involved in study design and drafting the manuscript. All authors participated in analysis and interpretation of data, intellectual content, draft revision, and approval of the final manuscript.

## Pre-publication history

The pre-publication history for this paper can be accessed here:

http://www.biomedcentral.com/1472-6947/12/49/prepub

## Supplementary Material

Additional file 1 **Appendix A: Division of 39 journals into development and validation sets.** Appendix B: Methods used to determine article relevance to glomerular disease. Click here for file
