# Supplementary material for: Glomerular disease search filters for Pubmed, Ovid Medline, and Embase: a development and validation study
Source: BMC Med Inform Decis Mak. 2012 Jun 6;12:49. doi: 10.1186/1472-6947-12-49 (PMC3471011; doi:10.1186/1472-6947-12-49)
Supplement: Additional file 1 — Appendix A: Division of 39 journals into development and validation sets. Appendix B: Methods used to determine article relevance to glomerular disease. [file 1472-6947-12-49-S1.docx]

Appendix A: Division of 39 journals into development and validation sets

| Rank | Journal | Number of Articles Contributed (total = 22,992)  n | Number of Articles with Glomerular Disease Content  n (%) |
| --- | --- | --- | --- |
| Development set | | | |
| 1 | American Journal of Clinical Oncology | 134 | 0 |
| 2 | American Journal of Clinical Pathology | 244 | 1 |
| 3 | American Journal of Kidney Diseases | 376 | 60 |
| 4 | American Journal of Transplantation | 462 | 6 |
| 5 | Annals of Internal Medicine | 562 | 3 |
| 6 | Archives of Disease in Childhood | 417 | 1 |
| 7 | BMJ | 2194 | 3 |
| 8 | Bone Marrow Transplantation | 358 | 1 |
| 9 | Calcified Tissue International | 112 | 0 |
| 10 | Clinical Pharmacology & Therapeutics | 147 | 1 |
| 11 | Diabetes Care | 703 | 27 |
| 12 | Diabetes/Metabolism Research Reviews | 66 | 3 |
| 13 | Investigative Radiology | 124 | 0 |
| 14 | Journal of Human Genetics | 183 | 1 |
| 15 | Journal of Hypertension | 445 | 8 |
| 16 | Journal of Infection | 258 | 2 |
| 17 | Journal of Trauma-Injury Infection & Critical Care | 663 | 0 |
| 18 | Journal of Urology | 1564 | 2 |
| 19 | Journal of Vascular Surgery | 600 | 0 |
| 20 | Journal of Viral Hepatitis | 130 | 0 |
| 21 | Kidney International | 888 | 152 |
| 22 | Nephrology Dialysis Transplantation | 856 | 125 |
| 23 | Netherlands Journal of Medicine | 116 | 1 |
| 24 | New England Journal of Medicine | 1949 | 5 |
| 25 | Pediatric Nephrology | 365 | 91 |
| 26 | Transplantation | 703 | 6 |
| **Total** | | **14619** | **499 (3.41 %)** |
| Validation set | | | |
| 1 | American Journal of Medicine | 487 | 8 |
| 2 | Annals of Thoracic Surgery | 1328 | 0 |
| 3 | Clinical Transplantation | 162 | 3 |
| 4 | Diabetic Medicine | 273 | 12 |
| 5 | Family Practice | 103 | 0 |
| 6 | Journal of Pediatrics | 520 | 2 |
| 7 | Journal of the American Society of Nephrology | 502 | 101 |
| 8 | Lancet | 1796 | 2 |
| 9 | Peritoneal Dialysis International | 177 | 1 |
| 10 | Radiology | 586 | 1 |
| 11 | Surgical Endoscopy | 453 | 0 |
| 12 | Transplantation Proceedings | 1168 | 10 |
| 13 | Urology | 818 | 1 |
| **Total** | | **8373** | **141 (1.68 %)** |

Appendix B: Methods used to determine article relevance to glomerular disease

| 1. | Full-text versions of articles were reviewed. |
| --- | --- |
| 2. | Terms used to define glomerular disease content are listed below. |
| 3. | The article is relevant if: glomerular disease content is the article’s main purpose/focus/patient population. Glomerular disease was defined as any disease in which the glomerulus of the kidney is affected, resulting in hyperplasia, atrophy, necrosis, scarring, or deposits in the glomeruli.   \| a. \| Regarding focus: for basic science papers, the article is relevant if the aim of the article is to elucidate processes related to glomerular disease. \| \| --- \| --- \| \| b. \| Regarding patient population: the article is relevant if EITHER at least 30% of included patients are associated with glomerular disease; OR 50 or more patients are associated with glomerular disease. \| |
| 4. | The article is not relevant if: glomerular disease content is only mentioned in reference to patient exclusion criteria. |

| Terms which define glomerular disease content: |
| --- |
| Inclusions |
| Glomerular diseases • Glomerulonephropathy • Glomerulopathy • Glomerulonephritis • Glomerulosclerosis • Glomerular diseases • Glomerular dysfunction • Glomerulonephritides • Nephropathy |
| Nephrotic/Nephritic syndrome • Hereditary nephritis • Balkan nephropathy • Benign familial hematuria |
| Biopsy classification of glomerulonephritis • Anti-GBM antibody disease • Anti-glomerular basement membrane disease • Goodpasture’s syndrome • Balkan nephropathy • Bright’s disease • Benign familial hematuria • Collapsing glomerulosclerosis • Focal (segmental) glomerulosclerosis • Glomerular hematuria • Hereditary nephritis • IgA nephropathy (aka Berger’s disease) • Membranoproliferative glomerulonephritis • Membranous nephropathy • Mesangiocapillary glomerulonephritis • Minimal change disease • Post-infectious glomerulonephritis • Post-streptococcal glomerulonephritis • Proliferative glomerulonephritis • Thin basement membrane disease • Immune complex nephritis • Lupus nephritis/glomerulonephritis • Lupoid nephritis/glomerulonephritis • Lupus nephropathy • Diabetic nephropathy/glomerulosclerosis/nephrosclerosis/glomerulopathy • Kinnelstiel-Wilson disease/syndrome/nephropathy • Nodular/intercapillary glomerulosclerosis • HIV-associated nephropathy |
| Exclusions |
| Chronic allograft nephropathy |
